# The association between the diabetes mellitus duration and the risk of pulmonary tuberculosis in the elderly: evidence from a community-based study in China

**DOI:** 10.7189/jogh.16.04039

**Published:** 2026-05-08

**Authors:** Yijun He, Yan Qian, Henan Xin, Jiang Du, Xuefang Cao, Juanjuan Huang, Lingyu Shen, Yuanzhi Di, Yaqi Zhao, Zihan Li, Boxuan Feng, Jianguo Liang, Qi Jin, Ping Zhu, Lei Gao

**Affiliations:** 1 NHC Key Laboratory of Systems Biology of Pathogens, National Institute of Pathogen Biology, and Center for Tuberculosis Research, Chinese Academy of Medical Sciences and Peking Union Medical College, Beijing, China; 2 Key Laboratory of Pathogen Infection Prevention and Control (Ministry of Education), National Institute of Pathogen Biology, Chinese Academy of Medical Sciences and Peking Union Medical College, Beijing, China; 3 State Key Laboratory of Respiratory Health and Multimorbidity, National Institute of Pathogen Biology, Chinese Academy of Medical Sciences and Peking Union Medical College, Beijing, China; 4 Longyou County People's Hospital, Zhejiang, China; 5 Center for Diseases Control and Prevention of Quzhou, Zhejiang, China

## Abstract

**Background:**

Diabetes mellitus (DM) is one of the risk factors for pulmonary tuberculosis (TB) development, and the dual burden of these diseases poses a significant public health challenge. However, the impact of DM duration on the risk of pulmonary TB, which is crucial for developing precise intervention strategies, has not been clarified.

**Methods:**

We conducted a retrospective study based on an active case-finding project on pulmonary TB in Quzhou City, Zhejiang Province, in 2022, to explore the association between DM duration and the risk of pulmonary TB among elderly individuals aged ≥65 years. We followed participants from the 2022 baseline screening until the date of pulmonary TB diagnosis, death, or 31 December 2024, whichever came first.

**Results:**

Among the 212 616 participants with a median age of 72 (interquartile range = 68–76) years, 724 cases with pulmonary TB were reported during the follow-up of 521 173.131 person-years, with an incidence of 138.917/100 000 person-years in 2022–2024. Participants with DM had a significantly higher risk of developing pulmonary TB compared with those without DM (adjusted sub-distribution hazard ratio (aSDHR) = 1.353; 95% confidence interval (CI) = 1.062–1.724). Notably, participants with a DM duration of 2–5 years had a significantly higher risk of pulmonary TB (aSDHR = 2.125; 95% CI = 1.481–3.047), with no other DM duration groups showing significant associations.

**Conclusions:**

We found DM to be a risk factor for the development of pulmonary TB, with a particularly pronounced effect in the subgroup with a disease duration of 2–5 years. Further research is needed to verify this association, elucidate its underlying pathophysiological mechanisms, and conduct targeted cost-benefit analyses to provide a basis for public health decision-making.

Diabetes mellitus (DM) is a complex multi-system metabolic disorder characterised by hyperglycaemia, which can lead to complications, reduce quality of life, and increase mortality [1]. It significantly affects older adults aged ≥65 years, who account for nearly half of the affected population [2]. China, which is estimated to have the largest number of DM cases [3], is projected to be home to 54.3 million DM patients aged ≥65 years by 2030, with this number expected to further increase to 78.1 million by 2045 [4]. In addition, the condition has been shown to be an important risk factor for active tuberculosis (TB), latent TB infection (LTBI), the development of multidrug-resistant TB, and adverse TB treatment outcomes [5–10]. According to the latest global TB report from the World Health Organization, China ranks fourth in the world in terms of TB burden, with 696 000 newly diagnosed cases recorded in 2024 [11]. Although significant progress has been made in reducing the global TB burden over the past decade, it has been the slowest among older adults [12].

As people age, the risk of functional decline and coexistence of multiple diseases increases, and the susceptibility of DM patients to TB is also affected by multiple factors [13,14]. Having DM can significantly affect the number and functioning of innate immune cells, including monocyte differentiation, macrophage phagocytosis, and natural killer cell activity. T cells and B cells, for example, can specifically respond to exogenous antigens. The function of adaptive immune cells can be also affected by hyperglycaemia. Relatedly, TB may also cause glucose intolerance and worsen glycaemic control in DM patients [14–16]. The double burden of TB and DM has become a major public health challenge.

As a response strategy, community-wide screening for pulmonary TB, if conducted annually with an accurate screening test and high coverage, has been shown to reduce TB prevalence [17]. Yet implementing TB prevention and control policies across the large population of DM patients will be challenging, especially as there is no consensus on the effect of DM duration on the incidence risk of TB [18–20]. Therefore, based on a community-based active cases finding (ACF) project of pulmonary TB in the elderly aged ≥65 years in Quzhou City, we aimed to explore the association between DM duration and the risk of pulmonary TB to inform the formulation of precise intervention strategies.

## METHODS

### Study design, setting, and participants

We conducted this retrospective study within the framework of a community-based ACF project of pulmonary TB among the elderly aged ≥65 years in rural areas of Quzhou City, China. The target population for the ACF project were all persons aged ≥65 years residing in the rural areas of Quzhou City. Physicians performed physical examinations on each participant, while trained medical technicians collected fasting venous blood samples under sterile conditions and transported the samples to the laboratory for biochemical testing. In addition, they performed chest x-ray examinations of each participant and referred them with radiographic abnormalities consistent with active pulmonary TB identified during the screening process to the local hospital for further examination. The diagnosis of pulmonary TB was performed in accordance with the national health industry standard for pulmonary TB diagnosis (WS 288-2017) [21]. We reported this study in accordance with the STROBE guidelines (Table S1 in the **Online Supplementary Document**) [22].

### Study design and participants

We used a retrospective study design based on the community-based ACF project for pulmonary TB, with 2022 annual screening as the baseline. To obtain the occurrence of pulmonary TB diagnosis, we cross-matched the 2022 baseline screening database with the local TB information management system, which is connected to the national electronic system that systematically records the information of TB cases [23], assigning them a unique ID number in the process. In addition, to obtain participants' DM diagnosis dates (recorded on or before 2022), we extracted the first DM diagnosis date from the local chronic disease management system, managed by public health institutions, and cross-matched it with the baseline screening database using a unique ID number. We collected the date of death from the Cause of Death Registration Reporting Information System and cross-matched it with the baseline screening database using unique ID numbers.

For each participant, we calculated the follow-up time from the date of the 2022 baseline screening until the earliest of the following events: pulmonary TB (confirmed either clinically or microbiologically), death, or the study end date (31 December 2024), whichever came first. For the pulmonary TB events, the diagnosis date was the first time that they were diagnosed with pulmonary TB during the follow-up period. We calculated the DM duration as the interval between the date of the first-recorded DM diagnosis and the date of the 2022 baseline screening. Lastly, we calculated body mass index (BMI) as weight in kg divided by height in m^2^ (kg/m^2^).

We included participants aged ≥65 years who participated in the 2022 baseline screening project in Quzhou City and had complete identity and demographic information. We excluded individuals diagnosed with pulmonary TB within 90 days before or after 2022 baseline screening, those identified as suspected pulmonary TB during 2022 baseline screening, individuals with DM without a recorded diagnosis date, and those diagnosed with DM in 2022 after baseline screening, as well as participants with a follow-up time of ≤90 days.

### Statistical analysis

We used the Kolmogorov-Smirnov test to assess the distribution of continuous variables, non-normally distributed variables were reported as medians and interquartile ranges, and the Wilcoxon rank-sum test to compare their distribution. We otherwise expressed categorical variables as frequencies and percentages, and used Pearson χ^2^ test to compare their distribution.

We verified the proportional hazards assumption using Schoenfeld residuals, and for variables that violated it, added time-dependent interaction terms (variable × log (follow-up time)) to the model. According to the assessments, we used either Cox proportional hazards regression models or non-proportional hazards regression models to estimate hazard ratios (HRs) with 95% confidence intervals (CIs) to evaluate the association between independent variables and the risk of pulmonary TB. To account for the competing risk of death, we additionally employed the Fine-Gray sub-distribution hazard regression models. Using the same covariates as those included in the Cox models, we estimated the sub-distribution HRs (SDHR) with their corresponding 95% CIs. In addition, we plotted the cumulative incidence curves, which were estimated using Fine-Gray models with death treated as a competing event.

To assess the dose-response relationship between continuous DM duration (years) and the risk of pulmonary TB, we fitted a Cox model using three-knot restricted cubic splines to detect potential nonlinear associations. We used participants with 0 years of DM duration (non-DM participants) as the reference. To assess potential effect modification of the association between DM duration and the risk of pulmonary TB, we conducted subgroup analyses stratified by four clinically relevant factors: fasting blood glucose (FBG) level (<7.0 *vs.* ≥7.0 mmol/L), age (≤71 *vs.* ≥72 years), sex (male *vs.* female), and BMI (<24.0 *vs.* ≥24.0 kg/m^2^). To determine whether the observed association was confounded by potential misclassification of DM status and to verify the stability of the primary findings across groups with distinct metabolic profiles, we conducted sensitivity analyses using stratified exclusion criteria based on DM status and FBG levels. We performed four analyses: exclusion of non-DM participants with FBG < 7.0 mmol/L, exclusion of non-DM participants with FBG≥7.0 mmol/L, exclusion of DM participants with FBG < 7.0 mmol/L, and exclusion of DM participants with FBG ≥ 7.0 mmol/L.

We used SAS, version 9.4 (SAS Institute, Cary, NC, USA) and *R*, version 4.4.2 (R Core Team, Vienna, Austria) for all analyses. The statistical tests were two-sided, and we defined statistical significance as *P* < 0.05.

## RESULTS

We included 212 616 participants in the analysis (Figure S1 in the **Online Supplementary Document**). They had a median age of 72 years (interquartile range = 68–76), 53.72% were female, and 55.90% had a BMI of 18.5–24.0 kg/m^2^. Among participants with DM, 46.60% exhibited a FBG ≥ 7.0 mmol/L (**Table 1**). Overall, during the observation period of 521 173.131 person-years from 2022 to 2024, 8169 participants died, and 724 cases of pulmonary TB were recorded; the incidence rate of pulmonary TB, therefore, was 138.917/100 000 person-years. Participants with DM had a significantly higher risk of developing pulmonary TB compared with those without DM, with an adjusted SDHR (aSDHR) of 1.353 (95% CI = 1.062–1.724) (**Table 2**; Figure S2 in the **Online Supplementary Document**). Among participants with BMI<24.0 kg/m^2^, those with DM had a significantly higher risk of pulmonary TB compared to non-DM participants (aSDHR = 1.462; 95% CI = 1.097–1.948). However, there was no significant association between DM and pulmonary TB incidence risk in participants with BMI≥24.0 kg/m^2^ (aSDHR = 1.452; 95% CI = 0.963–2.189) (Table S2 in the **Online Supplementary Document**).

**Table 1:** Baseline characteristics of the participants in Quzhou, 2022

		DM, n (%)		
	**n (%)**	**No**	**Yes**	***P*-value**
**Total**	212 616 (100.00)	187 852 (100.00)	24 764 (100.00)	
**Age, MD (IQR)**	72 (68–76)	72 (68–76)	72 (68–75)	<0.001*
**Age group, in years**				<0.001†
65–69	74 043 (34.82)	65 484 (34.86)	8559 (34.56)	
70–74	71 055 (33.42)	62 380 (33.21)	8675 (35.03)	
75–79	38 596 (18.15)	33 826 (18.01)	4770 (19.26)	
≥80	28 922 (13.60)	26 162 (13.93)	2760 (11.15)	
**Gender**				<0.001†
Male	98 392 (46.28)	89 793 (47.80)	8599 (34.72)	
Female	114 224 (53.72)	98 059 (52.20)	16 165 (65.28)	
**BMI, in kg/m^2^**				<0.001†
<18.5	14 880 (7.00)	14 102 (7.51)	778 (3.14)	
18.5–24.0	118 853 (55.90)	107 224 (57.08)	11 629 (46.96)	
24.0–28.0	62 878 (29.57)	53 584 (28.52)	9294 (37.53)	
≥28.0	16 005 (7.53)	12 942 (6.89)	3063 (12.37)	
**FBG level, in mmol/L**				<0.001†
<7.0	188 128 (88.48)	174 905 (93.11)	13 223 (53.40)	
≥7.0	24 488 (11.52)	12 947 (6.89)	11 541 (46.60)	
**TC level, in mmol/L**				0.006†
<6.2	190 048 (89.39)	168 039 (89.45)	22 009 (88.87)	
≥6.2	22 568 (10.61)	19 813 (10.55)	2755 (11.13)	
**TG level, in mmol/L**				<0.001†
<2.3	183 260 (86.19)	163 917 (87.26)	19 343 (78.11)	
≥2.3	29 356 (13.81)	23 935 (12.74)	5421 (21.89)	

BMI – body mass index, DM – diabetes mellitus, FBG – fasting blood glucose, IQR – interquartile range, MD – median, TC – total cholesterol, TG – triglycerides

*Wilcoxon rank-sum test.

†Pearson χ^2^ test.

**Table 2:** Association analysis on the risk of pulmonary TB in Quzhou, 2022–2024

	IR of pulmonary TB, n/N (%)	Follow-up time (PY)	IR per 100 000 PY	Crude HR (95% CI)*	aHR (95% CI)†	aSDHR (95% CI)†
**Total**	724/212 616 (0.34)	521 173.131	138.917			
**Age group, in years**						
≤71	287/105 325 (0.27)	260 709.459	110.084	ref	ref	ref
≥72	437/107 291 (0.41)	260 463.672	167.778	1.529 (1.317–1.774)	1.376 (1.184–1.599)	1.342 (1.154–1.560)
**Gender**						
Male	517/ 98 392 (0.53)	239 476.505	215.888	2.940 (2.502–3.454)	2.848 (2.418–3.355)	2.815 (2.389–3.316)
Female	207/114 224 (0.18)	281 696.626	73.483	ref	ref	ref
**BMI, in kg/m^2^**						
<18.5	104/14 880 (0.70)	353 90.015	293.868	1.940 (1.567–2.402)	1.928 (1.555–2.391)	1.876 (1.513–2.327)
18.5–24.0	442/118 853 (0.37)	290 996.008	151.892	ref	ref	ref
24.0–28.0	155/62 878 (0.25)	155 216.369	99.861	0.657 (0.547–0.789)	0.693 (0.576–0.834)	0.698 (0.580–0.840)
≥28.0	23/16 005 (0.14)	39 570.738	58.124	0.383 (0.252–0.582)	0.445 (0.292–0.679)	0.447 (0.293–0.683)
**DM status**						
No	627/187 852 (0.33)	460 594.905	136.128	ref	ref	ref
Yes	97/24 764 (0.39)	60 578.226	160.124	1.178 (0.951–1.459)	1.360 (1.071–1.727)	1.353 (1.062–1.724)
**FBG level, in mmol/L**						
<7.0	616/188 128 (0.33)	461 248.633	133.551	ref	ref	ref
≥7.0	108/24 488 (0.44)	59 924.498	180.227	1.352 (1.102–1.659)	1.375 (1.096–1.726)	1.368 (1.087–1.723)
**TC level, in mmol/L**						
<6.2	665/190 048 (0.35)	465 166.659	142.960	ref	ref	ref
≥6.2	59/22 568 (0.26)	56 006.472	105.345	0.738 (0.565–0.963)	0.984 (0.751-1.289)	0.989 (0.759–1.288)
**TG level, in mmol/L**						
<2.3	661/183 260 (0.36)	448 945.910	147.234	ref	ref	ref
≥2.3	63/29 356 (0.21)	72 227.221	87.225	0.591 (0.457–0.766)	0.803 (0.615–1.048)	0.805 (0.621–1.042)

aHR – adjusted hazard ratio, aSDHR – adjusted sub-distribution hazard ratio, BMI – body mass index, CI – confidence interval, DM – diabetes mellitus, FBG – fasting blood glucose, HR –hazard ratio, IR – incidence rate, PY – person years, ref – reference, TB – tuberculosis, TC – total cholesterol, TG – triglycerides

*Cox models with no covariates adjusted for.

†Cox models and Fine-Gray models with all variables incorporated into the models.

When stratified into two groups by FBG level, participants with FBG ≥ 7.0 mmol/L had a significantly higher risk of developing pulmonary TB, with an aSDHR of 1.368 (95% CI = 1.087–1.723) (**Table 2**). To address both DM status and FBG level, we further divided the participants into four groups. Compared to non-DM participants with FBG < 7.0 mmol/L, DM participants with FBG ≥ 7.0 mmol/L had the highest risk of pulmonary TB (aSDHR = 1.819; 95% CI = 1.221–2.710), while non-DM participants with FBG ≥ 7.0 mmol/L also had a significantly elevated risk (aSDHR = 1.486; 95% CI = 1.072–2.059) (Table S3 in the **Online Supplementary Document**).

**Figure 1:**
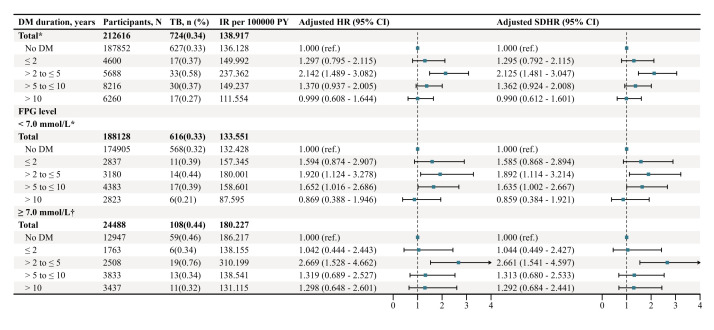
Subgroup analysis of the association between the DM duration and the risk of pulmonary TB in Quzhou, 2022–24. *We used Cox models and Fine-Gray models with adjustments for age, gender (categorical variable), BMI, FBG, TC, TG. †We used Cox models and Fine-Gray models with adjustments for age, gender (categorical variable), BMI, FBG, TC, TG, DM duration × log (follow-up time), and TC × log (follow-up time). aHR – adjusted hazard ratio, aSDHR – adjusted sub-distribution hazard ratio, BMI – body mass index, CI – confidence interval, DM – diabetes mellitus, FBG –fasting blood glucose, IR – incidence rate, PY – person years, TB – tuberculosis, TC – total cholesterol, TG – triglycerides.

There was a significant nonlinear association between continuous DM duration and the risk of pulmonary TB (*P* for nonlinearity = 0.001) (Figure S3 in the **Online Supplementary Document**). We then further divided participants with DM into four groups based on DM duration: ≤2 years, 2–5 years, 5–10 years, and >10 years (**Table 3**; Figure S4 in the **Online Supplementary Document**). Participants with a DM duration of 2–5 years had a significantly higher risk of developing pulmonary TB than those without DM (aSDHR = 2.125, 95% CI = 1.481–3.047), whereas no other DM duration groups showed significant associations. To further explore the heterogeneity of the association between DM duration and the risk of pulmonary TB, we performed subgroup analysis stratified by age, gender, BMI, and FBG level. Across all subgroups, participants with a DM duration of 2–5 years consistently had a significantly higher risk of pulmonary TB compared to those without DM (**Figure 1**; Table S4 in the **Online Supplementary Document**).

**Table 3:** Baseline characteristics of the mass screening participants stratified by DM duration in Quzhou, 2022

		Participants with DM stratified by DM duration in years, n (%)				
	**No DM, n (%)**	**≤2**	**2–5**	**5–10**	**>10**	*P*-value*
**Total**	187 852 (100.00)	4600 (100.00)	5688 (100.00)	8216 (100.00)	6260 (100.00)	
**Age group, in years**						<0.001
≤71	92 973 (49.49)	2467 (53.63)	3018 (53.06)	4113 (50.06)	2754 (43.99)	
≥72	94 879 (50.51)	2133 (46.37)	2670 (46.94)	4103 (49.94)	3506 (56.01)	
**Gender**						<0.001
Male	89 793 (47.80)	1947 (42.33)	2154 (37.87)	2717 (33.07)	1781 (28.45)	
Female	98 059 (52.20)	2653 (57.67)	3534 (62.13)	5499 (66.93)	4479 (71.55)	
**BMI, in kg/m^2^**						<0.001
<18.5	14 102 (7.51)	126 (2.74)	161 (2.83)	246 (2.99)	245 (3.91)	
18.5–24.0	107 224 (57.08)	2069 (44.98)	2505 (44.04)	3875 (47.16)	3180 (50.80)	
24.0–28.0	53 584 (28.52)	1775 (38.59)	2215 (38.94)	3119 (37.96)	2185 (34.90)	
≥28.0	12 942 (6.89)	630 (13.70)	807 (14.19)	976 (11.88)	650 (10.38)	
**FBG level, in mmol/L**						<0.001
<7.0	174 905 (93.11)	2837 (61.67)	3180 (55.91)	4383 (53.35)	2823 (45.10)	
≥7.0	12 947 (6.89)	1763 (38.33)	2508 (44.09)	3833 (46.65)	3437 (54.90)	
**TC level, in mmol/L**						<0.001
<6.2	168 039 (89.45)	4155 (90.33)	5114 (89.91)	7219 (87.87)	5521 (88.19)	
≥6.2	19 813 (10.55)	445 (9.67)	574 (10.09)	997 (12.13)	739 (11.81)	
**TG level, in mmol/L**						<0.001
<2.3	163 917 (87.26)	3610 (78.48)	4355 (76.56)	6364 (77.46)	5014 (80.10)	
≥2.3	23 935 (12.74)	990 (21.52)	1333 (23.44)	1852 (22.54)	1246 (19.90)	

BMI – body mass index, DM – diabetes mellitus, FBG – fasting blood glucose, TC – total cholesterol, TG – triglycerides

*Pearson χ^2^ test.

**Figure 2:**
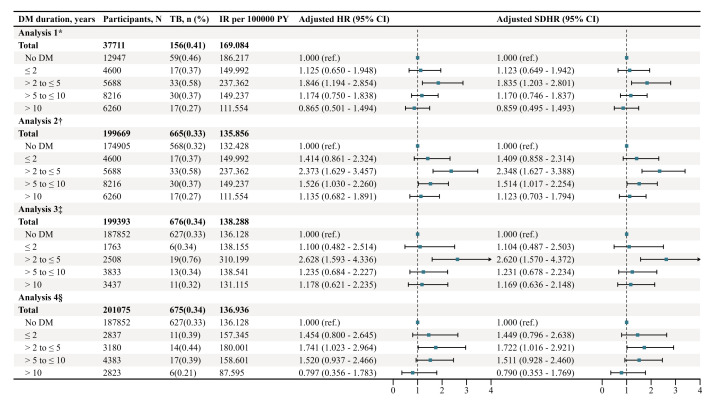
Sensitivity analysis of the association between the DM duration and the risk of pulmonary TB in Quzhou, 2022–24. *Exclusion of non-DM participants with FBG < 7.0 mmol/L. We used Cox models and Fine-Gray models with adjustments for age, gender (categorical variable), BMI, FBG, TC, TG, and gender × log (follow-up time). †Exclusion of non-DM participants with FBG ≥ 7.0 mmol/L. We used Cox models and Fine-Gray models with adjustments for age, gender (categorical variable), BMI, FBG, TC, and TG. ‡Exclusion of DM participants with FBG < 7.0 mmol/L. We used Cox models and Fine-Gray models with adjustments for age, gender (categorical variable), BMI, FBG, TC, TG, and age × log (follow-up time). §Exclusion of DM participants with FBG ≥ 7.0 mmol/L. We used Cox models and Fine-Gray models with adjustments for age, gender (categorical variable), BMI, FBG, TC, TG, age × log (follow-up time), and FBG × log (follow-up time). aHR – adjusted hazard ratio, aSDHR – adjusted sub-distribution hazard ratio, BMI – body mass index, CI – confidence interval, DM – diabetes mellitus, FBG – fasting blood glucose, IR – incidence rate, PY – person years, TB – tuberculosis, TC – total cholesterol, TG – triglycerides.

To evaluate the robustness of the association between DM duration and the risk of pulmonary TB, we performed a sensitivity analysis using stratified exclusion criteria by DM status and FBG levels (**Figure 2**). In the first analysis (excluding non-DM participants with FBG < 7.0 mmol/L), participants with DM duration of 2–5 years had a significantly higher risk of pulmonary TB than non-DM participants (aSDHR = 1.835; 95% CI = 1.203–2.801), with no other DM duration groups showing significant associations. Consistent results were observed both in the third (excluding DM participants with FBG < 7.0 mmol/L) and the fourth analysis (excluding DM participants with FBG ≥ 7.0 mmol/L). In second analysis (excluding non-DM participants with FBG ≥ 7.0 mmol/L), both duration groups of 2–5 years (aSDHR = 2.348; 95% CI = 1.627–3.388) and 5–10 years (aSDHR = 1.514; 95% CI = 1.017–2.254) were associated with a significantly higher risk of pulmonary TB compared with those without DM.

## DISCUSSION

Our findings indicate that DM is associated with an increased risk of pulmonary TB, and that participants with a DM duration of 2–5 years had a significantly higher risk of developing pulmonary TB than those without DM. This may provide new insights and practical references for implementing precise pulmonary TB screening and intervention strategies for people with DM in the community.

The immune response to MTB infection is highly complex, with TB progression resulting from dynamic interactions among the environment, the host, and the pathogen [24,25]. A systematic review and meta-analysis [8] have shown that patients with DM have a 1.5-fold increased risk of developing active TB compared to those without DM, which aligns with our findings. Multiple factors contribute to the increased susceptibility of patients with DM to TB, including direct effects related to hyperglycaemia and insulin resistance, as well as indirect effects on macrophage and lymphocyte function [15,26]. Simultaneously, DM may create an environment that facilitates the conversion of LTBI into active TB disease [14]. However, the exact mechanism linking DM and TB remains poorly understood, and further studies are warranted.

Imbalanced glycaemic control has been shown to impair immune responses to MTB infection [16]. We divided participants into two groups based on their baseline FBG levels. In both Cox and Fine-Gray models, participants with FBG ≥ 7.0 mmol/L had a higher risk of developing pulmonary TB than participants with FBG<7.0 mmol/L. Non-DM participants with FBG ≥ 7.0 mmol/L had a significantly higher risk of pulmonary TB than those non-DM participants with FBG < 7.0 mmol/L. This, to some extent, highlights the potential role of elevated FBG levels in the pathogenesis of pulmonary TB. In a cohort study of the elderly participants aged ≥65 years, participants with DM who had a baseline haemoglobin A1c ≥7% (*vs.* <7%) had significantly higher risks of active TB, culture-confirmed TB, and pulmonary TB [27]. However, the association between glycaemic control and the incidence risk of TB remains inconsistent across studies [27–31]. Since we considered only participants' baseline FBG levels and did not assess changes in FBG, further studies should explore whether dynamic blood glucose levels impact on the risk of pulmonary TB. This will provide a more accurate understanding of the relationship between blood glucose levels and the incidence of pulmonary TB, offering better references for clinical practice.

The association between DM duration and the risk of TB is a complex issue that has been explored in various studies across different populations, yielding inconsistent results [18–20]. A study conducted among Korean adults found that the risk of TB incidence increased with DM duration for new-onset DM (adjusted HR (aHR) = 1.32; 95% CI = 1.23–1.42), DM duration <5 years (aHR = 1.45; 95% CI = 1.36–1.54), and DM duration ≥5 years (aHR = 1.57; 95% CI = 1.48–1.66) [19]. However, a study with 15 years of follow-up in Denmark reported that the TB incidence rate decreased as DM duration increased, mainly during the first two years after DM diagnosis [18]. In our study, participants with a DM duration of 2–5 years had a significantly higher risk of developing pulmonary TB compared to those without DM, and this association remained stable when stratified by age, gender, BMI, and FBG level.

Lifestyle modifications that promote weight loss and improve insulin sensitivity, including diet and physical activity, may initially control blood glucose. However, over time, insulin secretion capacity decreases, and most people with type 2 DM will require pharmacologic therapy to achieve metabolic control [32]. A retrospective database analysis of 24 192 Chinese patients with type 2 DM demonstrated that only 30.9% of patients were adhered to insulin therapy, with a mean medication possession ratio of 0.499. During the follow-up period, about 53.0% of patients persisted on insulin therapy, while the mean time to non-persistence was 230.3 days [33]. Therefore, we hypothesise that newly diagnosed DM patients typically have high initial disease awareness and strong treatment compliance, enabling them to maintain disease at a relatively optimal level through lifestyle interventions or initial pharmacotherapy. Under such conditions, key immune components critical for anti-TB defence may remain relatively intact. Consequently, their susceptibility to active TB may not increase significantly compared to those without DM. Another hypothesis is that the impaired immune response in patients with DM may accelerate the progression from LTBI to active TB, leading to a quicker depletion of the LTBI pool among the DM patients [18,26]. Therefore, with the prolonged duration of DM, the LTBI pool may have been largely exhausted. These hypotheses may explain the trend of an initial increase followed by a decrease, with a statistically significant association observed in the 2–5 years duration group. However, given that the pathophysiological mechanisms remain unclear, these hypotheses require further research to elucidate the biological processes linking DM duration with pulmonary TB.

Being underweight is a well-recognised risk factor for TB. An updated systematic literature review and dose-response meta-analysis have shown an inverse dose-response relationship between BMI and TB risk across all populations, ranging from underweight to obese (15.0–35.0 kg/m^2^) [34]. However, overweight and obesity are key determinants of DM, and DM is, in turn, a well-established risk factor for TB [10,35]. A prospective population-based cohort study conducted in China showed that among participants with BMI>24.0 kg/m^2^, the risk of developing TB was similar in patients with and without DM (*P* = 0.953). Conversely, among participants with BMI≤24.0 kg/m^2^, participants with DM had a higher risk of developing active TB than those without DM (*P* = 0.006) [36]. Similarly, we found that among participants with BMI<24.0 kg/m^2^, participants with DM had a significantly higher risk of developing pulmonary TB than those without DM. There was no significant association between DM and the risk of pulmonary TB among participants with BMI≥24.0 kg/m^2^. In addition, elderly people with DM frequently experience delayed diagnosis, along with a higher likelihood of developing DM complications and increased risks of myocardial infarction and end-stage renal disease than younger patients [37]. The Framingham Heart Study showed that the DM duration was significantly associated with an increased risk of coronary heart disease death in DM patients. Specifically, each decade of DM duration corresponds to an 86% higher ten-year risk of coronary heart disease death [38]. Also, a previous systematic review found age and sex to be the most important predictors of mortality in the elderly [39]. To address the potential confounding effects mentioned above, we conducted subgroup analyses stratified by clinically relevant factors, including age, gender, BMI, and FBG level. Across subgroups, participants with a DM duration of 2–5 years consistently had a significantly higher risk of pulmonary TB compared to those without DM. However, the observed correlation needs to be rigorously validated through further research. Interpreting the underlying pathophysiological mechanisms involved remains a key focus of future research, which will help reveal the true nature of this relationship and provide guidance for clinical practice.

Several potential limitations should be considered when interpreting our results. First, although studies have shown that DM can promote the progression of LTBI to active TB [14], we did not test the baseline LTBI. Second, although we adjusted for several important demographic covariates, we did not assess others that may have affected the results of our analysis (*e.g.* lifestyle habits, comorbid conditions, and the quality of DM management). Third, we determined the initial sample size primarily by the number of participants in the active screening programme, as this was a retrospective study and we did not conduct a prior sample size calculation. However, based on the 2020 Seventh National Population Census, the population aged ≥65 years in Quzhou City was 420 138. Therefore, we covered an estimated >50% of this demographic, which confers a certain level of representativeness to the sample. Additionally, given that the elderly may attribute nonspecific symptoms (*e.g.* fatigue, nocturia, weight loss, and blurred vision) to normal ageing [2], the potential for delayed DM diagnosis means that the interval from initial diagnosis may not reliably approximate the true duration of the disease process. Lastly, TB is a notifiable infectious disease in China, requiring mandatory reporting, leading to highly complete and accurate case identification. Since DM is not a notifiable disease, it may lead to missed cases. Consequently, our analysis may be subject to bias due to differences in disease reporting.

## CONCLUSIONS

We found that the duration of DM may affect the risk of pulmonary TB, with a particularly pronounced higher risk observed in the intermediate group with a duration of 2–5 years. Targeted screening and intervention strategies could, therefore, be implemented for the elderly with DM, especially those with such a duration of DM. However, further research is needed to validate this association, elucidate its underlying pathophysiological mechanisms, and conduct targeted cost-effectiveness analyses to provide more robust evidence for public health policies.

## Additional material


Online Supplementary Document
