# Supplementary material for: The association between the diabetes mellitus duration and the risk of pulmonary tuberculosis in the elderly: evidence from a community-based study in China
Source: J Glob Health. 2026 May 8;16:04039. doi: 10.7189/jogh.16.04039 (PMC13154338; doi:10.7189/jogh.16.04039)
Supplement: Online Supplementary Document [file jogh-16-04039-s001.pdf]

Supplement to: He Y, Qian Y, Xin H, Du J, Cao X, Huang J, Shen L, Di Y, Zhao Y, Li Z, Feng B, Liang J, Jin Q, Zhu P, Gao L. The association between the diabetes mellitus duration and the risk of pulmonary tuberculosis in the elderly: evidence from a community-based study in China. J Glob Health. 2026;16:04039.

Supplementary Table S1. STROBE Statement—Checklist of items that should be included in reports of ***cohort studies***

	Item No	Recommendation	Page No
**Title and abstract**	1	(*a*) Indicate the study's design with a commonly used term in the title or the abstract	1
		(*b*) Provide in the abstract an informative and balanced summary of what was done and what was found	1
**Introduction**			
Background/rationale	2	Explain the scientific background and rationale for the investigation being reported	1-2
Objectives	3	State specific objectives, including any prespecified hypotheses	2
**Methods**			
Study design	4	Present key elements of study design early in the paper	2
Setting	5	Describe the setting, locations, and relevant dates, including periods of recruitment, exposure, follow-up, and data collection	2
Participants	6	(*a*) Give the eligibility criteria, and the sources and methods of selection of participants. Describe methods of follow-up	3
		(*b*) For matched studies, give matching criteria and number of exposed and unexposed	NA
Variables	7	Clearly define all outcomes, exposures, predictors, potential confounders, and effect modifiers. Give diagnostic criteria, if applicable	3
Data sources/ measurement	8*	For each variable of interest, give sources of data and details of methods of assessment (measurement). Describe comparability of assessment methods if there is more than one group	2-3
Bias	9	Describe any efforts to address potential sources of bias	2-3
Study size	10	Explain how the study size was arrived at	9
Quantitative variables	11	Explain how quantitative variables were handled in the analyses. If applicable, describe which groupings were chosen and why	3
Statistical methods	12	(*a*) Describe all statistical methods, including those used to control for confounding	3
		(*b*) Describe any methods used to examine subgroups and interactions	3
		(*c*) Explain how missing data were addressed	3
		(*d*) If applicable, explain how loss to follow-up was addressed	3
		(*e*) Describe any sensitivity analyses	3
**Results**			
Participants	13*	(a) Report numbers of individuals at each stage of study—eg numbers potentially eligible, examined for eligibility, confirmed eligible, included in the study, completing follow-up, and analysed	Supplementary
		(b) Give reasons for non-participation at each stage	Supplementary

		(c) Consider use of a flow diagram	Supplementary
Descriptive data	14*	(a) Give characteristics of study participants (eg demographic, clinical, social) and information on exposures and potential confounders	4-6
		(b) Indicate number of participants with missing data for each variable of interest	4-6
		(c) Summarise follow-up time (eg, average and total amount)	4-6
Outcome data	15*	Report numbers of outcome events or summary measures over time	Supplementary
Main results	16	(*a*) Give unadjusted estimates and, if applicable, confounder-adjusted estimates and their precision (eg, 95% confidence interval). Make clear which confounders were adjusted for and why they were included	5
		(*b*) Report category boundaries when continuous variables were categorized	4
		(*c*) If relevant, consider translating estimates of relative risk into absolute risk for a meaningful time period	NA
Other analyses	17	Report other analyses done—eg analyses of subgroups and interactions, and sensitivity analyses	6-7
**Discussion**			
Key results	18	Summarise key results with reference to study objectives	6
Limitations	19	Discuss limitations of the study, taking into account sources of potential bias or imprecision. Discuss both direction and magnitude of any potential bias	9
Interpretation	20	Give a cautious overall interpretation of results considering objectives, limitations, multiplicity of analyses, results from similar studies, and other relevant evidence	9
Generalisability	21	Discuss the generalisability (external validity) of the study results	9
**Other information**			
Funding	22	Give the source of funding and the role of the funders for the present study and, if applicable, for the original study on which the present article is based	9

*Give information separately for exposed and unexposed groups.

Note: An Explanation and Elaboration article discusses each checklist item and gives methodological background and published examples of transparent reporting. The STROBE checklist is best used in conjunction with this article (freely available on th e Web sites of PLoS Medicine at http://www.plosmedicine.org/, Annals of Internal Medicine at http://www.annals.org/, and Epidemiology at http://www.epidem.com/). Information on the STROBE Initiative is available at http:// www.strobe-statement.org.

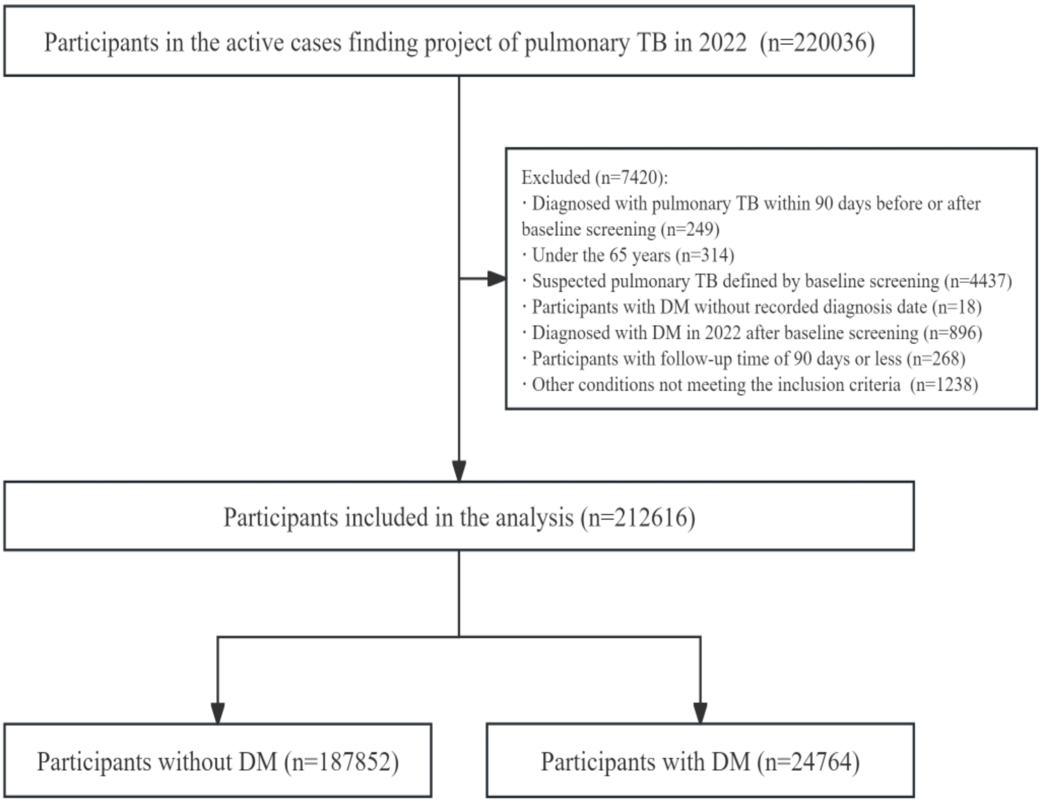

Supplementary Figure S1. Flowchart of the participants in Quzhou, 2022. DM – diabetes mellitus; TB – tuberculosis.

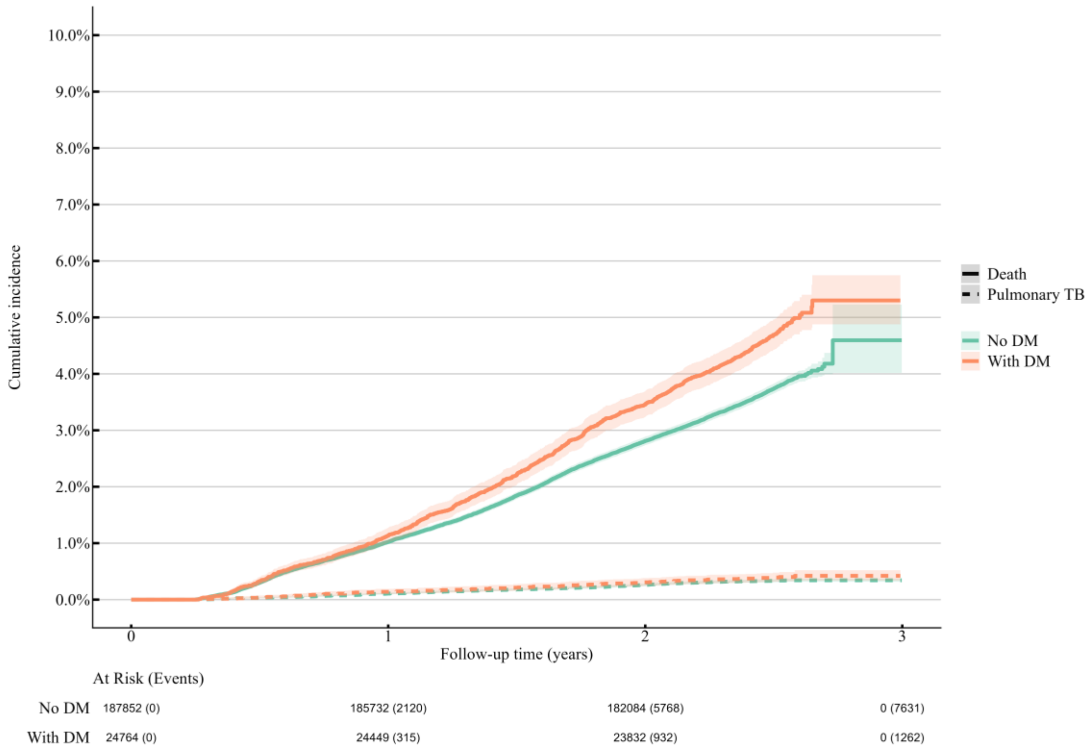

Supplementary Figure S2. Cumulative incidence of pulmonary tuberculosis stratified by diabetes mellitus status.

Cumulative incidence was estimated based on Fine-Gray models, with death treated as a competing event. No covariates were adjusted in the model.

DM – diabetes mellitus; TB – tuberculosis.

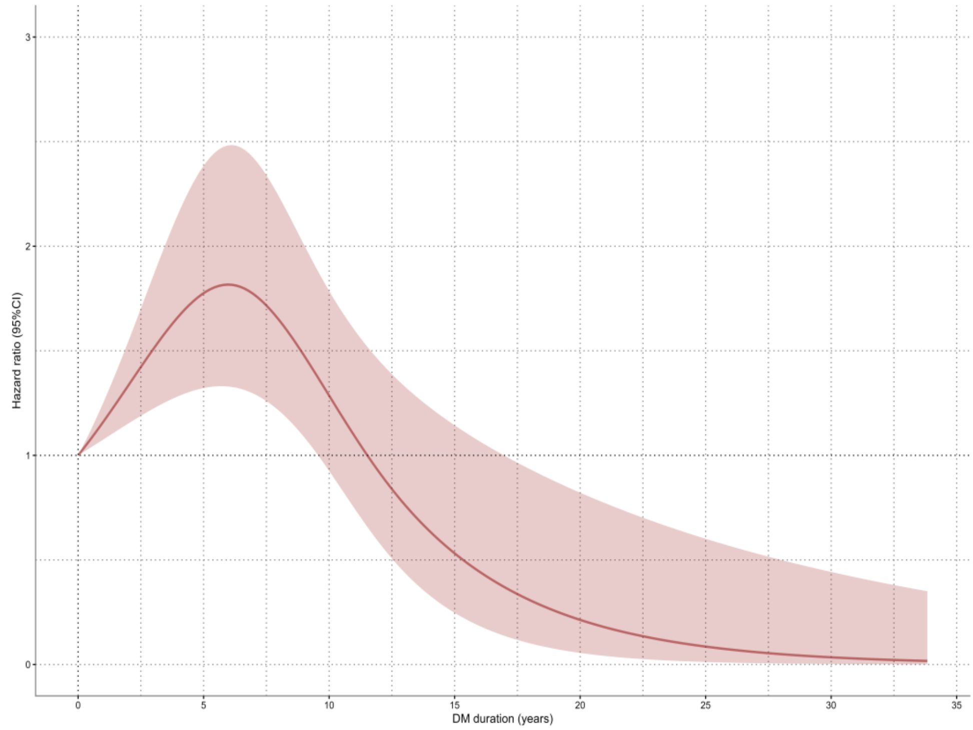

Supplementary Figure S3. Dose-response association between diabetes mellitus duration and the risk of pulmonary tuberculosis in Quzhou, 2022-2024. The Cox model was adjusted for age, gender (categorical variable), body mass index, fasting blood glucose, total cholesterol, and triglycerides. The red curve and shaded area represent the predicted hazard ratio and 95% confidence interval, respectively. The reference for diabetes mellitus duration is 0 years (i.e., no diabetes mellitus) (*P* for nonlinearity =0.001).

CI – confidence interval.

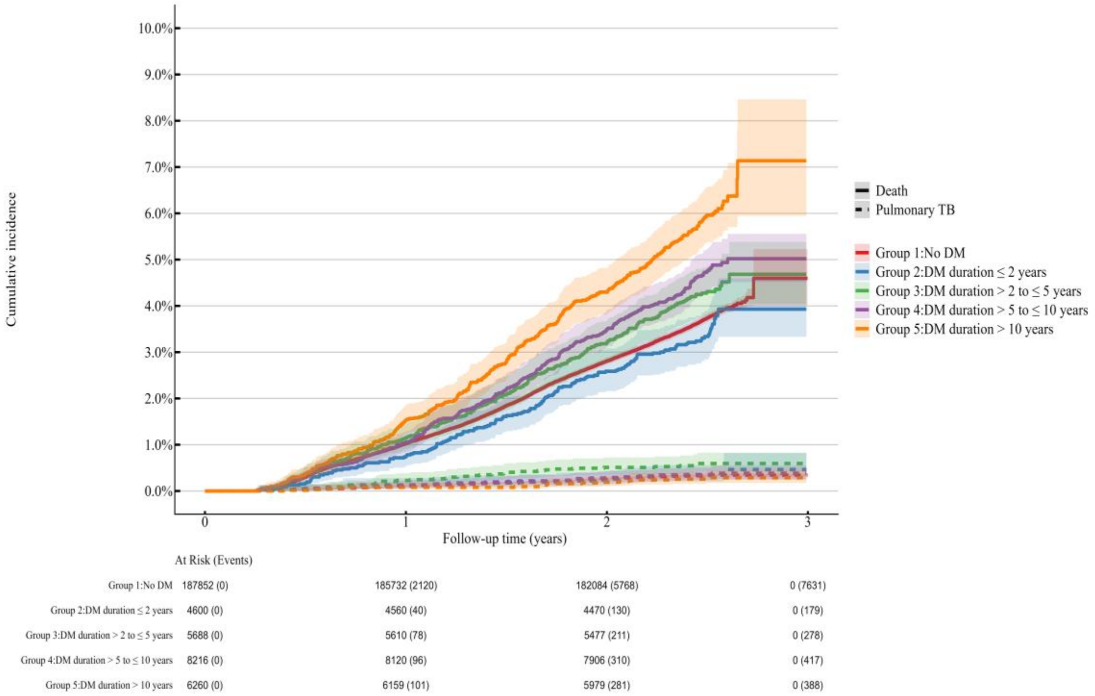

Supplementary Figure S4. Cumulative incidence of pulmonary tuberculosis stratified by diabetes mellitus duration.

Cumulative incidence was estimated based on Fine-Gray models, with death treated as a competing event. No covariates were adjusted in the model.

DM – diabetes mellitus; TB – tuberculosis.

Supplementary Table S2. Risk of pulmonary tuberculosis stratified by body mass index.

DM status	BMI < 24.0 kg/m^2 (N= 133733)*		BMI ≥ 24.0 kg/m^2 (N= 78883)†	
	aHR (95% CI)	aSDHR (95% CI)	aHR (95% CI)	aSDHR (95% CI)
No	1.000 (ref.)	1.000 (ref.)	1.000 (ref.)	1.000 (ref.)
Yes	1.474 (1.103- 1.970)	1.462 (1.097- 1.948)	1.457 (0.966- 2.197)	1.452 (0.963- 2.189)

aHR – adjusted hazard ratio, aSDHR – adjusted sub-distribution hazard ratio, BMI – body mass index; CI – confidence interval; DM – diabetes mellitus.

*Cox models and Fine–Gray models were used, with adjustments for age, gender (categorical variable), body mass index, fasting blood glucose, total cholesterol, triglycerides, and age× log (follow-up time).

†Cox models and Fine–Gray models were used, with adjustments for age, gender (categorical variable), body mass index, fasting blood glucose, total cholesterol, and triglycerides.

Supplementary Table S3. Diabetes mellitus status and fasting blood glucose level on the risk of pulmonary tuberculosis in Quzhou, 2022-2024.

DM status	FBG level, mmol/L	IR of pulmonary TB, n/N (%)	IR per 100000 PY	aHR (95% CI)*	aSDHR (95% CI)*
No	< 7.0	568/ 174905 (0.32)	132.428	1.000 (ref.)	1.000 (ref.)
No	≥ 7.0	59/ 12947 (0.46)	186.217	1.493 (1.061 -2.100)	1.486 (1.072 - 2.059)
Yes	< 7.0	48/ 13223 (0.36)	148.436	1.437 (1.059- 1.949)	1.426 (1.051 - 1.934)
Yes	≥ 7.0	49/ 11541 (0.42)	173.506	1.829 (1.214 - 2.754)	1.819 (1.221 - 2.710)

aHR – adjusted hazard ratio, aSDHR – adjusted sub-distribution hazard ratio, CI – confidence interval; DM – diabetes mellitus; FBG – fasting blood glucose; IR – incidence rate; PY – person years; TB – tuberculosis.

*Cox models and Fine-Gray models were used, with adjustments for age, gender (categorical variable), body mass index, fasting blood glucose, total cholesterol, triglycerides, and DM_FBG × log (follow-up time). The DM_FBG variable was constructed based on DM status and FBG level, and was divided into 4 groups.

Supplementary Table S4. Subgroup analysis of the association between the diabetes mellitus duration and the risk of pulmonary tuberculosis in Quzhou, 2022-2024.

DM duration, years	Participants, N	TB, n (%)	IR per 100000 PY	aHR (95% CI)	aSDHR (95% CI)
Age group, years					
≤ 71*					
No DM	92973	243(0.26)	105.565	1.000 (ref.)	1.000 (ref.)
≤2	2467	11(0.45)	179.423	1.948 (1.049 -3.617)	1.950 (1.040 -3.655)
2–5	3018	15(0.50)	201.644	2.240 (1.298 -3.867)	2.231 (1.297 -3.838)
5–10	4113	12(0.29)	117.981	1.362 (0.746 -2.486)	1.353 (0.725 -2.523)
> 10	2754	6(0.22)	88.499	0.972 (0.422 -2.239)	0.967 (0.426 -2.195)
≥ 72†					
No DM	94879	384(0.40)	166.662	1.000 (ref.)	1.000 (ref.)
≤2	2133	6(0.28)	115.314	0.821 (0.364 -1.852)	0.818 (0.363 -1.842)
2–5	2670	18(0.67)	278.466	2.092 (1.283 -3.413)	2.070 (1.279 -3.348)
5–10	4103	18(0.44)	181.250	1.382 (0.846 -2.258)	1.371 (0.837 -2.246)
> 10	3506	11(0.31)	130.031	1.008 (0.542 -1.874)	0.995 (0.549 -1.802)
Gender					
Male‡					
No DM	89793	452(0.50)	206.769	1.000 (ref.)	1.000 (ref.)
≤2	1947	15(0.77)	315.053	1.666 (0.986 -2.815)	1.664 (0.982 -2.819)
2–5	2154	20(0.93)	384.013	1.992 (1.252 -3.169)	1.974 (1.246 -3.127)
5–10	2717	19(0.70)	288.219	1.436 (0.892 -2.311)	1.431 (0.886 -2.309)
> 10	1781	11(0.62)	255.001	1.201 (0.650 -2.218)	1.187 (0.643 -2.192)
Female§					

No DM	98059	175(0.18)	72.316	1.000 (ref.)	1.000 (ref.)
≤2	2653	2(0.08)	30.428	0.507 (0.125 -2.059)	0.506 (0.124 -2.065)
2–5	3534	13(0.37)	149.517	2.503 (1.391 -4.504)	2.489 (1.395 -4.441)
5–10	5499	11(0.20)	81.421	1.317 (0.698 -2.483)	1.306 (0.670 -2.543)
> 10	4479	6(0.13)	54.917	0.825 (0.354 -1.919)	0.815 (0.396 -1.681)
BMI, kg/m^2					
< 24.0¶					
No DM	121326	483(0.40)	163.042	1.000 (ref.)	1.000 (ref.)
≤2	2195	11(0.50)	204.735	1.376 (0.750 -2.523)	1.371 (0.748 -2.512)
2–5	2666	16(0.60)	246.777	1.711 (1.024 -2.857)	1.697 (1.023 -2.816)
5–10	4121	24(0.58)	239.710	1.727 (1.127 -2.646)	1.712 (1.108 -2.644)
> 10	3425	12(0.35)	145.015	1.029 (0.570 -1.858)	1.015 (0.572 -1.803)
≥ 24.0†					
No DM	66526	144(0.22)	87.617	1.000 (ref.)	1.000 (ref.)
≤2	2405	6(0.25)	100.652	1.198 (0.524 -2.740)	1.199 (0.517 -2.780)
2–5	3022	17(0.56)	229.134	2.880 (1.699 -4.882)	2.855 (1.680 -4.852)
5–10	4095	6(0.15)	59.464	0.789 (0.342 -1.821)	0.787 (0.342 -1.810)
> 10	2835	5(0.18)	71.796	0.992 (0.395 -2.490)	0.986 (0.410 -2.371)

aHR – adjusted hazard ratio, aSDHR – adjusted sub-distribution hazard ratio, CI – confidence interval; BMI – body mass index; DM – diabetes mellitus; IR – incidence rate; PY – person years; TB – tuberculosis.

* Cox models and Fine–Gray models were used, with adjustments for age, gender (categorical variable), body mass index, fasting blood glucose, total cholesterol, triglycerides, age× log (follow-up time), and fasting blood glucose× log (follow-up time).

†Cox models and Fine–Gray models were used, with adjustments for age, gender (categorical variable), body mass index, fasting blood glucose, total cholesterol, and triglycerides.

‡Cox models and Fine–Gray models were used, with adjustments for age, body mass index, fasting blood glucose, total cholesterol, triglycerides, fasting blood glucose× log (follow-up time), and triglycerides× log (follow-up time).

§Cox models and Fine–Gray models were used, with adjustments for age, body mass index, fasting blood glucose, total cholesterol, and triglycerides.

¶Cox models and Fine–Gray models were used, with adjustments for age, gender (categorical variable), body mass index, fasting blood glucose, total cholesterol, triglycerides, and age× log (follow-up time).
